# Mucinous Colorectal Carcinoma in a 17-Year-Old Male: A Diagnosis with Low Clinical Index of Suspicion

**DOI:** 10.1155/2019/6371579

**Published:** 2019-09-25

**Authors:** James Joseph Yahaya, Edward Ketson Msokwa, Alex Mremi

**Affiliations:** ^1^Department of Biomedical Sciences, College of Health Sciences (CHS), The University of Dodoma, Dodoma, Tanzania; ^2^Department of Surgery, College of Health Sciences (CHS), The University of Dodoma, Dodoma, Tanzania; ^3^Department of Pathology, Kilimanjaro Christian Medical Centre (KCMC), Kilimanjaro, Tanzania

## Abstract

Colorectal carcinoma (CRC) is commonly found in adults. CRC in the pediatric population is extremely rare. Usually, CRC is diagnosed in children at advanced stage due to a low clinical index of suspicion. Mucinous type of CRC and its signet ring variant are the most common histological types which carry very poor clinical outcomes. This paper reports a 17-year-old male who presented with mild pallor and a 3-month history of abdominal pain accompanied with a mass on the left lower quadrant, and it was then confirmed histologically to be mucinous CRC of signet ring variant. This paper will help to raise awareness among the physicians and pediatricians in including CRC in the preliminary workouts for the purpose of shortening the delay for diagnosis which in turn would compromise the prognosis of the patients.

## 1. Introduction

Pediatric CRC has a poor prognosis compared with adult CRC mainly due to delayed diagnosis. Thus, early diagnosis based on a high degree of suspicion could be the most important factor in a more favorable prognosis, especially in patients with predisposing factors. CRC is one of the major causes of cancer-related death globally [1, 2]. The most common histological subtype of CRC is adenocarcinoma NOS, of which mucinous adenocarcinoma is a distinct subtype and is characterized by abundant mucinous components that comprise at least 50% of the tumour volume.

Mucinous CRC differs from other adenocarcinoma in terms of clinical and histopathological characteristics [3]. Mucinous CRC is found in 10%–20% of CRC patients and occurs more commonly in younger patients [4]. Furthermore, mucinous CRC is usually located more frequently in the proximal colon than in the distal part of the colon. Moreover, mucinous CRC is often diagnosed when it is already in advanced stages (stage III and IV), and it has poorer responses to chemotherapy as compared to the nonmucinous counterparts [5].

Signet ring cell carcinoma (SRCC) is defined by the presence of more than 50% of signet ring cells in the tumour, and more than 96% of it occurs in the stomach and the rest occur in the colon, rectum, gallbladder, pancreas, urinary bladder, and breast [6]. It has been reported that the frequency of SRCC occurring in the colon among adolescents and children is about 1% compared to 13% occurring in adults [6–8]. The impact on prognosis of SRCC has been found to be poorer due to the fact that it is diagnosed at the advanced clinical stage, distant lymph node metastasis, and distinct molecular patterns such as low rates of microsatellite, a high rate of BRAF gene mutations, and a low rate of KRAS gene mutations [9, 10].

We present a report of a case study of a 17-year-old male with a left-sided rectosigmoid CRC mucinous type of signet ring variant. The paper has also reviewed the details of the literature regarding different aspects of the disease in the general population and the pediatric population, in particular.

## 2. Case Presentation

A 17-year-old male was admitted to our hospital with a 3-month history of abdominal pain accompanied with a mass on the left lower quadrant which was associated with abdominal cramping, constipation, and passing of blood-stained stool per rectum. He denied history of diarrhea, heart beat awareness, and easy fatigability. In addition, he had no history of difficulty in breathing, vomiting, smoking, or drinking alcohol. He reported a long history of using herbs for the long-standing abdominal pain. His vital signs were as follows: blood pressure: 149/98 mmHg, heart rate: 106 beats per minute, oxygen saturation: 98%, and body temperature: 37.5°C. On examination, he was alert, mildly pale, and cachexic. Each abdominal examination showed mild abdominal distension with some traditional marks on both lateral sides. There was a firm and fixed mass in the left iliac fossa which was measured 8 × 6 × 3 cm. Digital rectal examination showed reduced sphincter tone and multiple firm masses palpable in the rectum with tenderness.

Ultrasound abdominal examination showed a heterogeneous mass measuring 5 × 5 cm in the left iliac fossa. Both kidneys had dilated calyces with moderate hydronephrosis. The hemoglobin level was 12 g/dl; serum sodium, potassium, and chloride were 130 mmol/L, 3.7 mmol/, and 97.7 mmol/L, respectively, and were all within the normal range. Other laboratory tests were creatinine: 113 *μ*mol/L; AST: 25 U/l; and ALT: 19 U/l. An abdominal computed tomography (CT) scan showed a circumferential tumour causing increasing of the thickness of the wall of the involved part of the intestine and measured 3.8 cm. The mass was arising from the rectum and extending to the sigmoid colon. There was luminal narrowing. However, there were no features of large-bowel obstruction. The rectal mass had a punctate calcification and had invaded the posterior wall of the urinary bladder. The mass was obstructing and encasing the distal ureters with resultant bilateral moderate hydroureteronephrosis. Gross free fluid was seen in the peritoneal cavity. All other abdominal organs were normal and in conclusion, the CT scan study of the abdomen showed a stage IIIC circumferential rectosigmoid tumour invading the posterior wall of the urinary bladder and encasing the distal ureter. There was no evidence of distant metastasis.

Based on the clinical history, physical examination, abdominal ultrasound, and CT scan, rectosigmoid tumour was given as a provisional diagnosis, and the patient was planned for exploratory laparotomy and biopsy. Intraoperatively, 1.5 litres of serous ascitic fluid was drained. There was a large tumour on the sigmoid, plastering posteriorly to the anterior abdominal wall. The ileum adhered to the tumour, but there was no obstruction. There was seedling in all the mesenteries and on the greater omentum. The caecum was also plastered posteriorly but patent. The tumour was graded as Dukes stage D because of involvement of the posterior wall of the urinary bladder as well as encasing the ureter. The tumour was inoperable. Loop transverse colostomy was raised at the left upper quadrant, and abdominal lavage with normal saline was done. After reviewing by a urologist, the patient was found to have obstructive hydronephrosis. Urethral diversion was done, but after assessment, the mass was found to have already encased the distal ureters. Incisional biopsies were taken from the greater omentum and rectum. Then, the patient was kept on antibiotics and analgesics.

**Figure 1:**
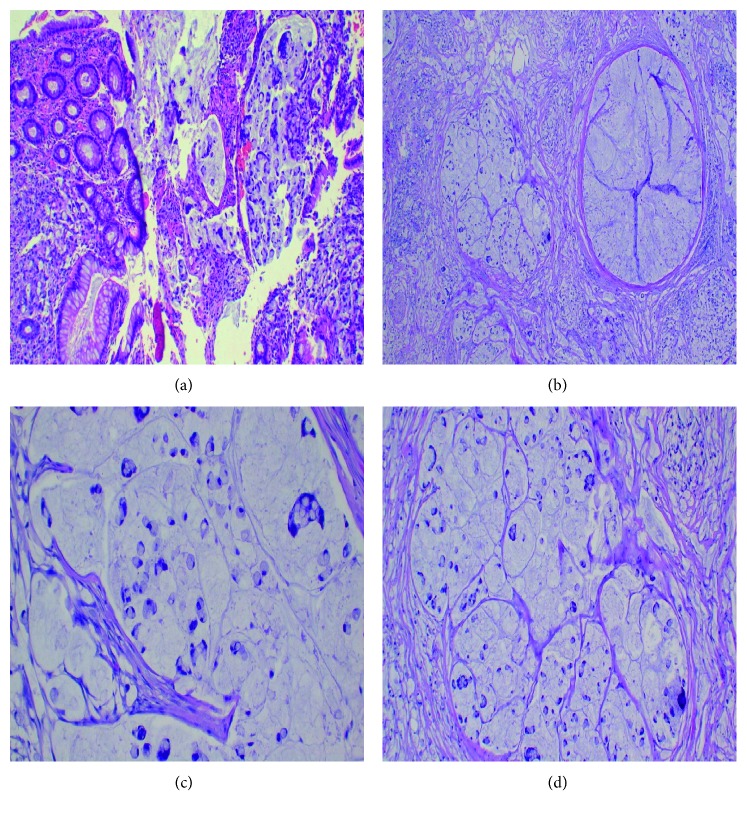
(a) Infiltrating tumour masses. The tumour has infiltrated through the mucosa. (b) The tumour masses have invaded the serosa. The mucin lakes are delineated by thick fibrous bands of tissue (H&E stains, ×40 magnification). (c, d) Infiltrating tumour cells. The tumour cells have signet ring shapes and are floating in lakes of mucin (H&E stains, ×400 magnification).

Microscopically, the tissue sections showed areas with mucosal infiltration of the tumour which was composed of lakes of mucin (Figure 1(a)), and the tumour had invaded to the level of serosa (Figure 1(b)). The tumour cells had abundant cytoplasm with hyperchomatic, pleomorphic, and densely stained nuclei which were pushed to the peripheral parts of the tumour cells giving the appearance of signet ring (Figure 1(c)), and in some areas the mucinous lakes were forming lobules (Figure 1(d)). In other areas, the tumour was surrounded by granulation tissue comprising of fibrosis, proliferating distended and congested blood vessels with sparse lymphocytes. Then, a histological diagnosis of mucinous adenocarcinoma of signet ring variant was made. Grade 4 was given (poorly differentiated) to the tumour.

After a week postoperatively, he developed convulsions and his condition became unstable. The renal function worsened. Haemodialysis before chemotherapy was advised. However, chemotherapy could not be initiated due to his unstable condition and renal insufficiency. The patient survived only for two months after diagnosis and died.

## 3. Discussion

Childhood CRC is a very rare disease entity. The incidence of CRC in children is 0.3 to 2 cases per 100, 000, making 0.4% of all malignant tumours with the highest mortality rate in patients below 15 years; however, most cases occur in the second decade of life [4, 5, 11]. There is a slight male predilection of CRC compared to females in the pediatric population, whereas in adults the sex distribution is equal [12].

The clinical signs and symptoms of CRC for children and adults are not different and are generally vague and not specific. Patients present with mild abdominal pain which is usually long standing, constipation, diarrhea, hematochezia, and weight loss. Other clinical features include alteration in bowel habit, tenesmus, anaemia, and loss of appetite [11–13]. Such clinical manifestations in children are normally underestimated. This greatly contributes to delayed diagnosis, hence leading to poor prognosis. Therefore, an effort must be made to educate health providers in the early recognition of this malignancy in children.

Majority of CRC patients have sporadic form of the disease. Familial CRC has been reported to be 20%–30% and 10% for adults and children, respectively [14]. The known genetic factors that can increase the risk of CRC at any age include Turcot's syndrome, ulcerative colitis (UC), familial occurrence of colorectal cancer, Bloom's syndrome, and polyposis syndromes. The polyposis syndromes which usually develop prior to the overt carcinoma include adenomatous polyps such as hereditary nonpolyposis CRC (HNPCC) or Lynch syndrome and familial adenomatous polyposis (FAP). Serrated (hyperplastic) and hamartomatous polyposis syndromes (juvenile polyposis syndrome and Peutz–Jegher's syndrome) have been also said to be genetically linked with CRC [12–15]. Other familial mismatch repair disorders predisposing to CRC are MUTYH-associated polyposis (MAP), attenuated FAP, Gardner syndrome-FAP with epidermoid cysts, osteomas, dental anomalies, and/or desmoid tumours [16].

The applicability of this model to children with CRC is unknown. However, there are some concerns which have been raised that suggest the possible difference in the way polyps develop into CRC between children and adults. Such existing differences are as follows: (1) the absence of CRC in children younger than 9 months since it takes approximately 10 years for CRC to develop; (2) premalignant adenomas are rarely seen in proximity to sporadic CRC in children; and (3) CRC in children tends to be of mucinous histology different from adults in which conventional adenocarcinoma is the most common type [5, 11, 13].

Colonoscopy is the gold standard of investigation during establishment of diagnosis of CRC in children and adults. Other advantages of this investigation include taking photographs and biopsy from the lesions. Other tests like stool for occult blood, barium studies, and CT colonography can be used in screening of the disease.

Surgical removal of the tumour has been advocated as the treatment of choice for both children and adults with CRC [16]. The principle for surgical approach for CRC in adults involves complete resection of the primary tumour (with minimum 5 cm free margin), its lymphatic bed, and any other involved organ(s). Because of limited prospective studies of surgical options of CRC in children due to rarity of the disease, resection follows adult guidelines [16]. Extent of colectomy depends on the location of the tumour, nature of the primary pathology, and the intent of the resection [17]. For the tumour at sigmoid colon, the entire sigmoid colon should be resected to the level of the peritoneal reflection and an anastomosis created between the descending colon and the upper rectum. After surgery, the prognosis is poor within the first 20 months; thereafter, the prognosis improves and becomes predictable [17].

Prognosis of the disease is influenced by factors such as aggressive histological subtypes (signet ring and mucinous adenocarcinoma), advanced tumour grade, and advanced stage of the disease [16, 17]. The role of adjuvant chemotherapy in improving survival seems to be not significant in both children and adults [10]. It was even once reported that adjuvant chemotherapy may sometimes compromise the clinical outcomes of the patients [17]. Thus, it is recommended as trial for those with poor prognosis [16]. Because of the small number of pediatric patients, treatment follows adult protocols. More studies have reported devastating clinical outcomes of CRC in the pediatric population. However, some studies have also reported the opposite results. For example, Sultan and associates [18] reported that the 5-year overall survival for children/adolescents and adults was 40% and 60%, respectively, whereas the 10-year overall survival for children/adolescents and adults was 31%, and 54%, respectively [18]. When determinants of high risk factors (HRFs) between children/adolescents and adults are evaluated, it has been found that children/adolescents carry more HRFs compared to adults. The HRFs in patients of CRC include tumour grade and stage and mucinous histological subtype [17, 18]. Such factors have been reported to have high hazard ratio (HR).

The current case had all the HRFs. Farner and colleagues [19] reported that patients with CRC who were aged <30 years had a relatively increased 5-year survival compared to the ones with the age between 30 and 40 years (*P*=0.02). Regarding the association of prognosis of CRC linked with polyposis syndromes such as APC and the CRC which is not linked with polyposis syndromes, studies have shown that CRC developing in children that is linked with polyposis syndromes has better prognosis unlike the one not linked with polyposis syndromes [16, 19]. Despite that the clinical symptoms of CRC for both children and adults are virtually similar, the prognosis in adults is better than that in children. This can be explained by the fact that CRC is less thought to be the differential among children as it is for adults. This contributes to the delay in diagnosis.

## 4. Conclusion

CRC in the pediatric population is very rare. Mucinous CRC is the most common histological type of CRC in the pediatric population with known poor prognosis. Almost every predictor of poor prognosis of CRC is found in pediatric patients as compared to the population of adult patients.
